# Clinical and financial impact of chronic kidney disease in emergency general surgery operations^☆^

**DOI:** 10.1016/j.sopen.2022.05.013

**Published:** 2022-06-07

**Authors:** Vishal Dobaria, Joseph Hadaya, Shannon Richardson, Cory Lee, Zachary Tran, Arjun Verma, Yas Sanaiha, Peyman Benharash

**Affiliations:** Cardiovascular Outcomes Research Laboratories (CORELAB), Division of Cardiac Surgery, University of California, Los Angeles David Geffen School of Medicine, Los Angeles, CA

## Abstract

**Introduction:**

Chronic kidney disease is frequently encountered in clinical practice and often requires more intricate management strategies. However, its impact on outcomes of patients warranting emergency general surgery has not been well characterized. The present study examined the association of chronic kidney disease stage on in-hospital outcomes and readmission following emergency general surgery using a nationally representative cohort.

**Methods:**

The 2016–2018 Nationwide Readmissions Database was queried to identify all adult hospitalizations for 1 of 6 common emergency general surgery operations. Patients were stratified by severity of chronic kidney disease into stages 1–3, stages 4–5, end-stage renal disease, and others (non*–*chronic kidney disease*)*. Regression models were used to examine factors associated with mortality, readmissions, and costs.

**Results:**

Of an estimated 985,101 patients undergoing emergency general surgery, 60,949 (6.2%) had a diagnosis of chronic kidney disease (1–3: 67.1%, 4–5: 11.5%, end-stage renal disease: 23.4%). Unadjusted rates of mortality increased with chronic kidney disease in a stepwise manner (2.1% in non*–*chronic kidney disease to 16.9 in end-stage renal disease, P < .001), as did 90-day readmissions (9.2% to 29.7%, respectively, P < .001). After adjustment, all stages of chronic kidney disease exhibited increases in risk-adjusted rates of mortality (range: 0.2% in chronic kidney disease 1–3 to 12.2% in end-stage renal disease, P < .001). Relative to non*–*chronic kidney disease, end-stage renal disease had the greatest cost burden for those undergoing small bowel resection (*β* +$83,600) and the least in cholecystectomy (+$30,400).

**Conclusion:**

Chronic kidney disease severity is associated with a stepwise increase in mortality, hospitalization costs, and 90-day readmissions. Our findings may better inform shared decision-making and have implications in benchmarking. Further studies for optimal management strategies in this high-risk group are needed.

## INTRODUCTION

Despite efforts to reduce the effects of hypertension and diabetes, the leading causes of kidney dysfunction in the United States, chronic kidney disease (CKD) continues to increase in prevalence. CKD is associated with significant health care expenditures and reduced quality of life, with end-stage renal disease alone costing more than $30 billion in excess costs for Medicare beneficiaries [1]. Furthermore, CKD is strongly associated with high rates of mortality and morbidity following a wide range of operative interventions [[2], [3], [4], [5], [6]].

Perioperative acute renal dysfunction has been associated with adverse events and prolonged recovery following major abdominal operations. In addition, emergency general surgery (EGS) operations pose a greater risk for deterioration of kidney function due to hypoperfusion, volume loss, and inflammation [7,8]. Moreover, clinical optimization and implementation of protective measures to reduce acute kidney injury (AKI) are often not feasible in the emergency setting. Nonetheless, it is now widely accepted that preexisting renal dysfunction is associated with greater risk of postoperative AKI [9,10].

Although limited studies have examined the impact of CKD on outcomes of appendectomy and operations for perforated ulcers, a systematic characterization of this association for all EGSs at the national level is lacking. Therefore, the present study used a contemporary national cohort to characterize the association of various stages of CKD on clinical outcomes and resource use following EGS.

## METHODS

### Study Design

The 2016–2018 Nationwide Readmissions Database (NRD) was queried to identify all adult (≥ 18 years) hospitalizations for any of the following 6 common EGS operations: large bowel resection, small bowel resection, cholecystectomy, repair of perforated ulcer, lysis of adhesions, and appendectomy. The NRD is the largest, all-payer database for national readmissions and is maintained by the Agency for Healthcare Research and Quality as part of the Healthcare Costs and Utilization Project. Using survey-weighted methodology, the NRD provides accurate estimates for approximately 60% of all US hospitalizations [11]. Patients are tracked across hospitalizations within each calendar year, facilitating study of readmissions.

Patients undergoing EGS within 2 days of nonelective admission were identified using relevant *International Classification of Diseases, 10^th^ Edition* (*ICD-10*) procedure codes as previously described [12]. Patients with indication for a trauma (4.4%) as well as those with missing entries for key data (2.5%) including age, sex, mortality, and costs were excluded. Patients were subsequently stratified by CKD stage using previously validated *ICD* diagnosis codes: non-CKD, CKD 1–3, CKD 4–5, and end-stage renal disease (ESRD) [2].

### Variables and Study Outcomes

Patient and hospital characteristics were reported as defined by the NRD Data Dictionary and included the following variables; age, sex, insurance status, annual household income quartile, hospital bed size, and hospital teaching status. The van Walraven modification of the Elixhauser Comorbidity Index was used to quantify the overall burden of comorbidities [13]. Specific patient comorbidities (Table 1) were further defined using *ICD-10* diagnosis codes. Overall costs for EGS hospitalizations were calculated by applying hospital-specific cost-to-charge ratios and adjusted for inflation using the 2018 Personal Health Care Index [14].

**Table 1:** Univariate patient and hospital characteristics across CKD groups

	*Non-CKD* *(*n *= 924,142)*	*CKD 1–3* *(*n *= 40,927)*	*CKD 4–5* *(*n *= 7030)*	*ESRD* *(*n *= 13,003)*	P *value*
Female (%)	60.6	45.9	51.1	45.7	<.001
Age (mean, SD)	55.1 ± 18.4	73.7 ± 11.7	73.4 ± 12.9	63.1 ± 14.1	<.001
Elixhauser Index (mean, SD)	2.3 ± 2.0	5.5 ± 2.0	5.8 ± 2.0	5.8 ± 2.0	<.001
Operative intervention (%)					<.001
Repair of perforated ulcer	2.0	2.9	3.2	3.2	
Appendectomy	4.9	2.8	3.4	4.7	
Lysis of adhesions	6.9	6.6	7.6	10.9	
Small bowel resection	9.0	13.7	15.1	16.4	
Large bowel resection	17.1	23.1	26.8	28.9	
Cholecystectomy	60.1	50.9	44.0	34.7	
Primary payer (%)					<.001
Medicare	36.7	80.2	79.7	76.3	
Medicaid	17.3	4.4	5.9	8.0	
Privately insured	36.7	12.6	11.7	13.3	
Other payer type	9.4	2.8	2.7	2.5	
Comorbidities (%)					
Cardiac valve disorder	2.8	10.1	10.8	8.2	<.001
Peripheral vascular disease	5.4	17.7	18.4	21.3	<.001
Congestive heart failure	5.4	27.7	36.9	32.5	<.001
Liver disease	7.7	8.9	8.6	15.2	<.001
Coronary artery disease	9.9	33.3	36.6	31.9	<.001
Arrhythmia	13.5	36.8	37.3	34.2	<.001
Chronic lung disease	14.7	24.8	24.3	20.8	<.001
Diabetes	16.8	44.4	49.4	54.9	<.001
Hypertension	45.2	88.9	89.4	88.9	<.001
Hospital bed size (%)					<.001
Small bed size	17.5	18.1	16.2	12.9	
Medium bed size	29.1	29.2	28.9	25.7	
Large bed size	53.4	52.7	54.9	61.3	
Teaching status (%)					<.001
Rural	27.8	26.9	26.2	22.8	
Metropolitan nonteaching	63.2	64.2	64.3	72.2	
Metropolitan teaching	9.0	8.9	9.5	4.9	

The primary outcomes of interests were in-hospital mortality and perioperative adverse events (AE). We defined AE as a composite of any of the following complications: cardiac (arrest and ventricular arrhythmias), respiratory (pneumonia, pneumothorax, acute respiratory distress syndrome, respiratory failure, prolonged mechanical ventilation), infectious (postoperative infection, and surgical site infection), cerebrovascular (stroke), and venous thromboembolism. Secondary outcomes included characterization of specific complications including AKI; hospitalization costs; nonhome discharge; and nonelective, 90-day readmissions.

### Statistical Analysis

Categorical variables were reported as percentage (%) and compared using the *χ*^2^ test. Continuous factors are reported as mean with standard deviation (SD) or median with interquartile ranges (IQRs) if non-normally distributed. Continuous variables were compared using the adjusted Wald and Mann–Whitney *U* tests, as appropriate. Multivariable regression models were developed to identify the independent association of covariates with outcomes of interest. Variable selection was performed using the least absolute shrinkage and selection operator. Briefly, the least absolute shrinkage and selection operator is a machine learning technique that reduces model overfitting and enhances out-of-sample validity for covariate selection [15]. Finally, models were evaluated using the receiver operator characteristic curve (ROC) or Akaike and Bayesian Information Criteria as appropriate. An interaction term between CKD category and EGS operative category was included to delineate the impact of CKD across operative subtypes on the study outcomes. Following regression, absolute risk-adjusted probabilities were calculated using the STATA *margins* command [16]. Regression outputs are reported as adjusted odds ratios (AORs) or beta coefficients (*β*s) with 95% confidence intervals (95% CIs) for logistic and linear regressions, respectively. The Kaplan–Meier method was used to study nonelective readmission at up to 90 days across groups. Data analysis was performed using Stata 16.0 (StataCorp, College Station, TX). The Institutional Review Board at the University of California, Los Angeles, deemed this study exempt from full review.

## RESULTS

### Cohort Characteristics

Of an estimated 985,101 adult EGS hospitalizations included for analysis, 60,949 (6.2%) had a diagnosis of CKD: CKD 1–3: 40,927 (67.1%), CKD 4–5: 7030 (11.5%), and ESRD: 13,003 (23.4%). ESRD patients more commonly underwent large bowel resection (30.0% vs 17.1, *P* < .001) and less commonly underwent cholecystectomy (17.1% vs 28.9%, *P* < .001) compared to non-CKD. Compared to those without CKD, patients with any diagnosis of CKD were more commonly older (71.4 ± 13.2 vs 55.0 ± 18.4, *P* < .001) and female (53.7% vs 39.4%, *P* < .001) and had greater mean Elixhauser Comorbidity Score (2.3 ± 2.0 vs 5.6 ± 2.0, *P* < .001). Patients with CKD 1–3, CKD 4–5, and ESRD had significantly greater rates of specific chronic conditions compared to non-CKD as seen in Table 1. Moreover, ESRD more commonly underwent operations at metropolitan, nonteaching centers (72.2%, *P* < .001) compared to other CKD and non-CKD groups (Table 1).

### Unadjusted Outcomes Following EGS by CKD Severity

Bivariate comparisons of outcomes across CKD severity groups are reported in Table 2. Patients in the CKD 1–3 group (63.1%) and CKD 4–5 group (48.0%) had higher rates of AKI following EGS compared to those without CKD (10.0%, *P* < .001). Unadjusted in-hospital mortality was significantly higher for patients with CKD 1–3 (5.9%), CKD 4–5 (9.5%), and ESRD (17.3%) compared to patients in the non-CKD group (2.1%, *P* < .001). Compared to others, ESRD patients had overall higher rates of in-hospital mortality after undergoing repair of perforated ulcer (34.3%), large bowel resection (29.6%) and small bowel resection (25.6%, *P* < .001 for all). Median index hospitalization costs paralleled CKD in a stepwise manner (Table 2) ranging from $13,500 in non-CKD (IQR $9,500–$20,800) to $30,800 for ESRD patients (IQR $17,200-$63,200).

**Table 2:** Univariate outcomes of patient characteristics across CKD groups

	*Non-CKD* *(*n *= 924,142)*	*CKD 1–3* *(*n *= 40,927)*	*CKD 4–5* *(*n *= 7030)*	*ESRD* *(*n *= 13,003)*	P *value*
Mortality (%)					
Overall	2.0	5.9	9.5	16.9	<.001
Cholecystectomy	0.2	1.1	2.8	4.6	<.001
Appendectomy	0.6	2.8	1.6	3.8	<.001
Lysis of adhesions	1.9	5.6	9.7	12.1	<.001
Small bowel resection	5.2	10.6	14.8	25.6	<.001
Large bowel resection	6.5	12.7	17.3	29.6	<.001
Repair of perforation	8.2	15.3	19.5	34.3	<.001

Complications (%)					
Adverse events	11.3	29.8	37.2	42.6	<.001
Acute kidney injury	10.0	48.0	63.1	¥	<.001
Respiratory	9.4	24.4	30.5	37.7	<.001
Cardiac	2.5	9.8	13.1	13.2	<.001
Venous thromboembolism	1.1	2.1	2.5	3.6	<.001
Infectious	0.8	1.3	1.1	2.4	<.001
Cerebrovascular	0.1	0.3	0.4	0.5	<.001

90-d readmissions (%)					
Overall	9.2	17.3	22.3	29.7	<.001
Cholecystectomy	6.1	13.3	19.5	28.7	<.001
Appendectomy	8.0	27.1	24.4	27.1	<.001
Lysis of adhesions	11.8	16.9	18.7	24.6	<.001
Repair of perforation	14.5	19.8	23.7	20.9	.007
Large bowel resection	14.7	20.2	21.1	22.6	<.001
Small bowel resection	14.9	19.6	19.5	22.0	<.001
Costs in $1000 (median, IQR)	13.5 (9.5–20.8)	20.1 (13.7–32.6)	22.8 (14.7–38.4)	30.8 (17.2–63.2)	<.001
Length of stay in days (median, IQR)	3 (2–6)	6 (3–10)	7 (4–12)	8 (4–16)	<.001

¥ Indicates omission due to collinearity.

**Fig 1:**
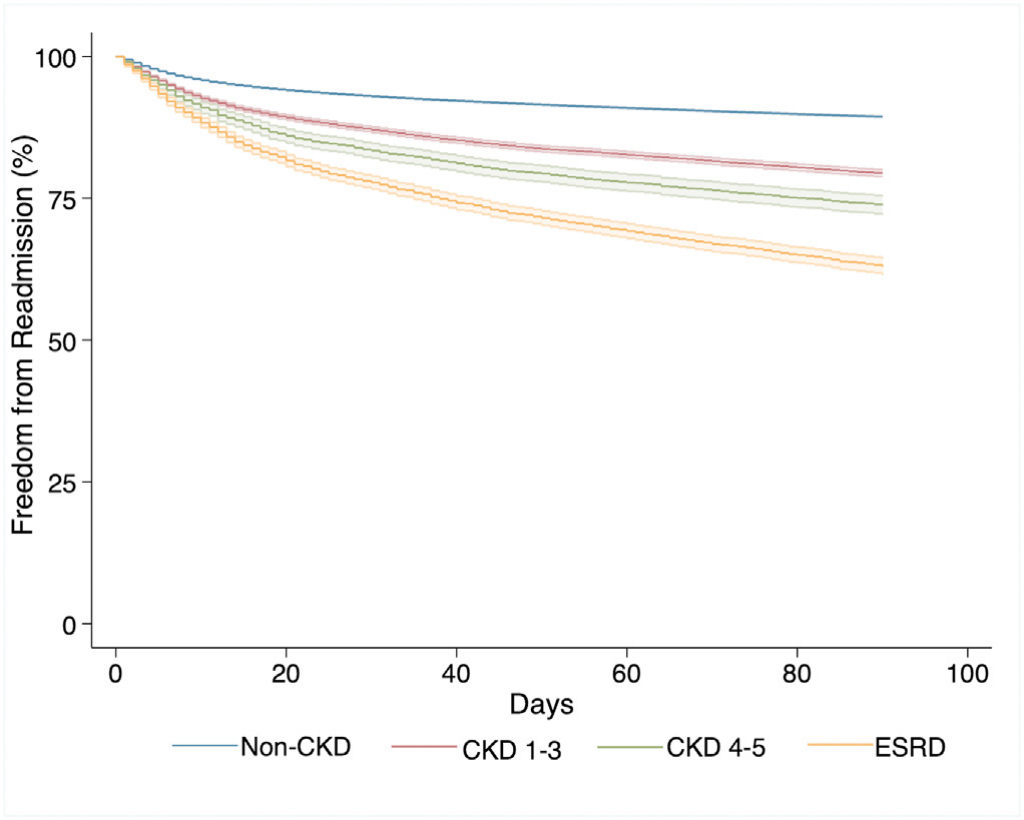
Kaplan–Meier estimate for readmission across CKD groups. Log-rank *P* < .001.

**Supplementary Fig 1:**
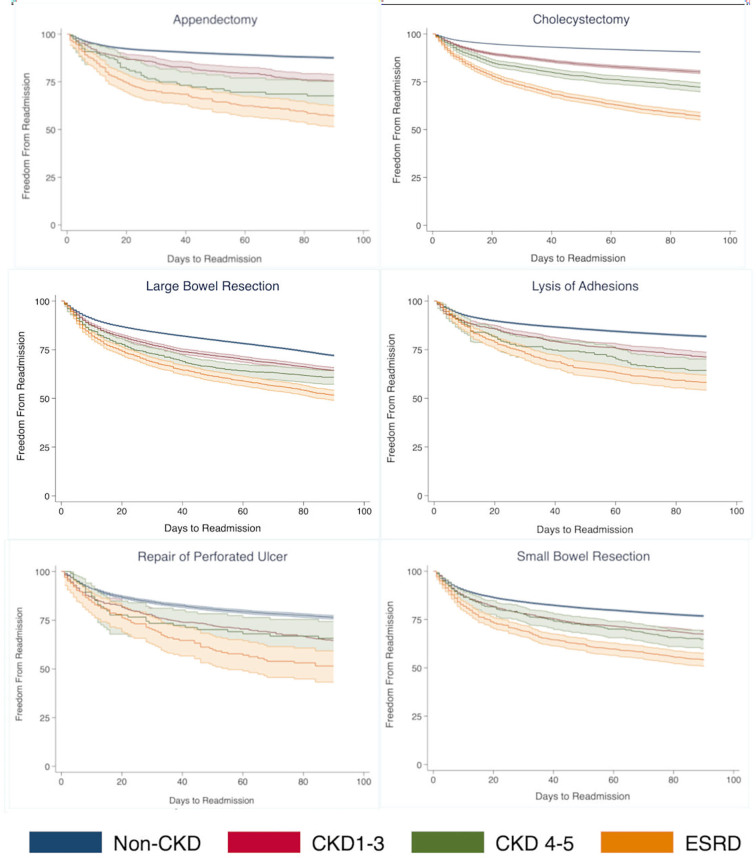
Readmissions across EGS subgroups with varying degrees of CKD.

Evaluation of readmissions at up to 90-days using Kaplan–Meier curves demonstrated a stepwise relationship between CKD severity and time to readmission (Fig 1). A similar trend of earlier readmission was observed when EGS was stratified by specific operation type (Supplementary Fig 1). Notably, ESRD patients undergoing cholecystectomy appeared to have the highest rates of readmission compared to others.

### Impact of CKD on Risk-Adjusted Outcomes Following EGS

**Fig 2:**
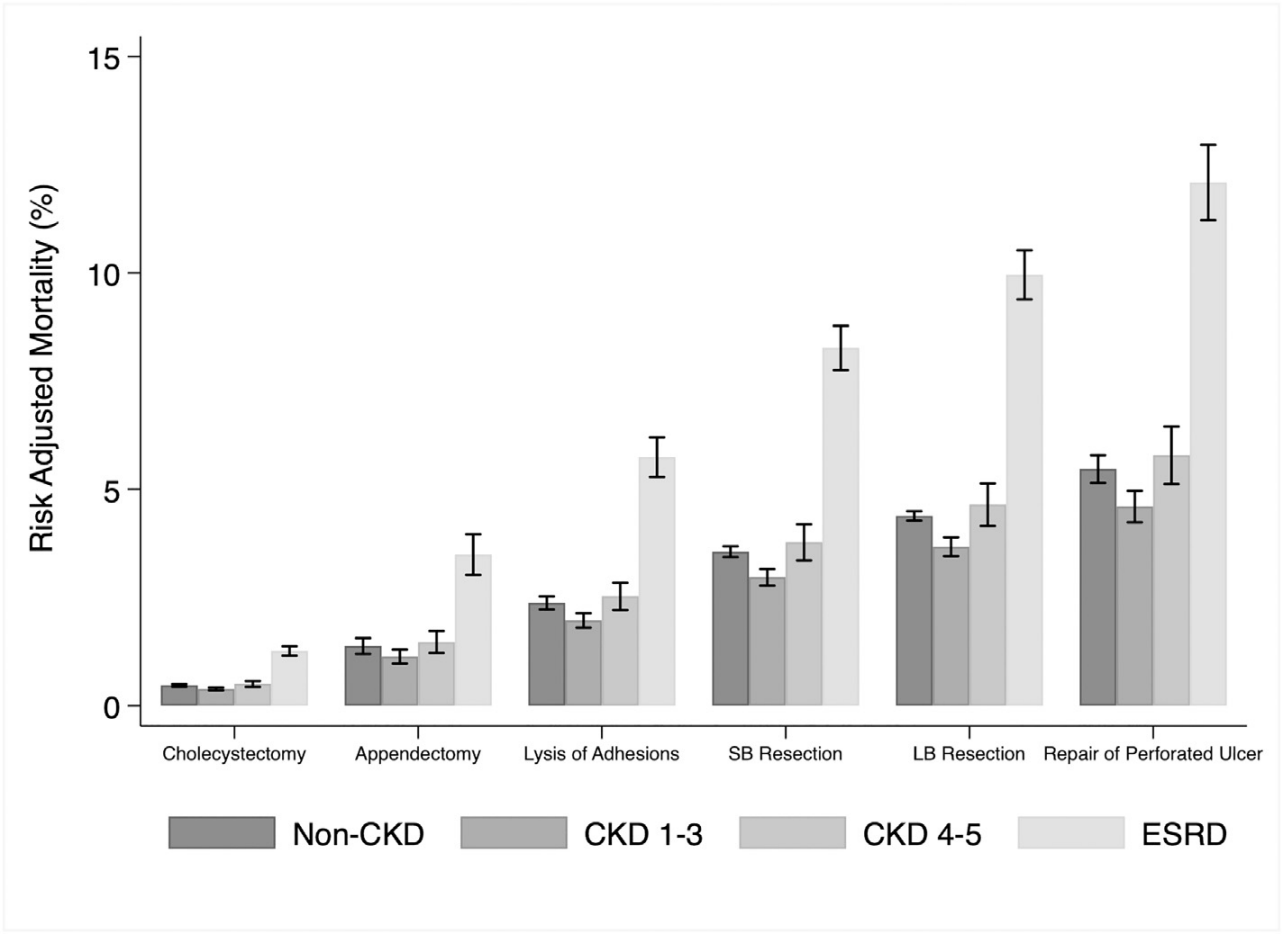
Risk-adjusted probabilities of mortality of CKD groups across EGS subtypes. *SB*, small bowel; *LB*, large bowel.

**Fig 3:**
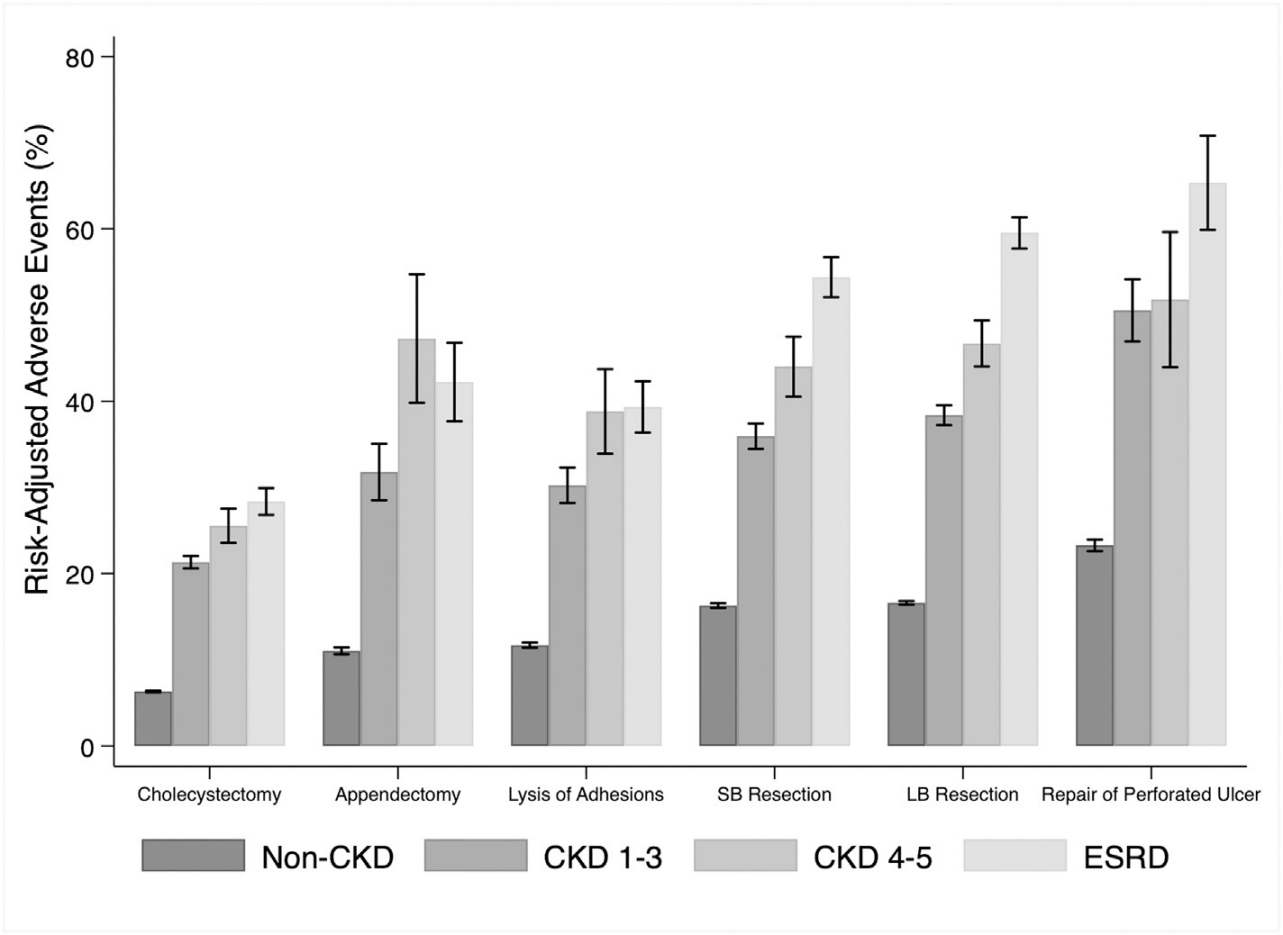
Risk-adjusted probabilities of AE of CKD groups across EGS subtypes. SB, small bowel; *LB*, large bowel.

Multivariable logistic and linear regression models were developed to assess associations between variables of interest and study outcomes. As shown in Table 3, ESRD was associated with significantly greater odds of mortality (AOR 2.85 95% CI 2.64–3.07 reference: non-CKD). Subset analyses were then performed to calculate the risk-adjusted probabilities of outcomes within each EGS operative category using the previous model. All CKD groups exhibited greater risk of mortality compared to non-CKD with ESRD conferring the greatest risk-adjusted rates (Fig 2). Notably, ESRD patients undergoing repair of perforated ulcers had the highest associated risk-adjusted mortality (12.2%) compared to non-CKD. As depicted in Figure 3, risk-adjusted rates of AE followed a similar stepwise trend as mortality.

**Table 3:** Association of covariates with in-hospital mortality (model *C*-statistic: = 0.72)

	*Odds ratio (95% CI)*	P *value*
Age (per 1-y increment)	1.04 (1.03–1.05)	<.001
Sex		
Male	Reference	
Female	1.03 (0.99–1.07)	.01
CKD severity		
Non‐CKD	Reference	
CKD 1–3	0.81 (0.76–0.87)	<.001
CKD 4–5	1.07 (0.94–1.22)	.28
ESRD	2.85 (2.64–3.07)	<.001

EGS operations		
Large bowel resection	Reference	
Small bowel resection	0.78 (0.74–0.82)	<.001
Cholecystectomy	0.08 (0.07–0.09)	<.001
Perforation repair	1.31 (1.21–1.41)	<.001
Lysis of adhesions	0.49 (0.45–0.53)	<.001
Appendectomy	0.27 (0.23–0.31)	<.001

Primary payer		
Privately insured	Reference	
Medicaid	1.23 (1.15–1.31)	<.001
Medicare	1.37 (1.25–1.49)	<.001
Other⁎	1.72 (1.56–1.91)	<.001
Elixhauser Comorbidity Index	1.42 (1.40–1.43)	<.001

Comorbidities		
Hypothyroidism	0.53 (0.50–0.56)	<.001
Coagulopathy	2.44 (2.33–2.56)	<.001
Diabetes	0.71 (0.67–0.74)	<.001
Peripheral vascular disease	1.38 (1.32–1.46)	<.001
Weight loss	0.97 (0.93–1.02)	.29
Hypertension	0.48 (0.46–0.50)	<.001
Anemia	0.43 (0.40–0.47)	<.001
Liver disease	2.40 (2.23–2.48)	<.001

Income quartiles		
76th–100th	Reference	
51st–75th	1.12 (1.06–1.19)	<.001
26th–50th	1.23 (1.16–1.30)	<.001
0th–26th	1.41 (1.33–1.49)	<.001

Hospital teaching Status		
Rural	Reference	
Urban nonteaching	1.11 (1.06–1.16)	<.001
Urban teaching	0.94 (0.86–1.02)	.13

⁎ Other is defined as reported by the NRD.

As seen in Table 4, relative to non-CKD, ESRD was associated with significantly greater odds of nonhome discharge (AOR: 1.59, 95% CI 1.49–1.69) as well as AE (AOR: 1.40, 95% CI 1.32–1.48) following EGS (reference: non-CKD for both). Additionally, both CKD 1–3 (AOR: 2.83, 95% CI 2.73–2.92, reference non-CKD) and CKD 4–5 *(*AOR: 4.96, 95% CI 4.60–5.35, reference: non-CKD) had increased odds of developing AKI. CKD 1–3 was also associated with greater odds of developing cerebrovascular complications (AOR: 1.20, 95% CI 1.05–1.91, reference non-CKD). Furthermore, ESRD were associated with increased odds of 90-day readmission (AOR 1.76, 95% CI 1.61–1.92), whereas CKD 1–3 (AOR: 0.98, 95% CI 0.92–1.05) and CKD 4–5 (AOR: 1.10, 95% CI 0.97–1.26) had comparable odds of readmission (ROC: 0.71).

**Table 4:** Adjusted odds ratios of CKD stages across complications

	*Non-CKD*	*CKD 1–3*	*CKD 4–5*	*ESRD*	*ROC*
Nonhome discharge	Ref	0.93 (0.90–0.96)	1.24 (1.14–1.34)	1.59 (1.49–1.69)	0.88
Complications					
Major adverse event	Ref	0.80 (0.77–0.83)	0.95 (0.88–0.98)	1.40 (1.32–1.48)	0.86
Infectious	Ref	0.94 (0.90–0.97)⁎	1.00 (0.93–1.08)	1.38 (1.31–1.45)⁎	0.71
Acute kidney injury	Ref	2.83 (2.73–2.92)⁎	4.96 (4.61–5.35)⁎	†	0.78
Respiratory	Ref	0.80 (0.76–0.83)⁎	0.93 (0.85–1.01)	1.47 (1.40–1.57)⁎	0.80
Cardiac	Ref	0.82 (0.65–0.79)⁎	1.00 (0.91–1.12)	1.15 (1.07–1.24)⁎	0.82
Venous thromboembolism	Ref	0.71 (0.63–0.80)⁎	0.64 (0.50–0.81)⁎	1.12 (0.98–1.29)	0.82
Cerebrovascular	Ref	1.20 (1.05–1.91)⁎	0.73 (0.23–2.33)	0.73 (0.31–1.68)	0.72
90-d readmissions	Ref	0.98 (0.92–1.05)	1.10 (0.97–1.26)	1.76 (1.61–1.92)⁎	0.71

⁎ Indicates statistical significance.

† Indicates omission due to collinearity.

Regression models were then developed to identify the independent association of CKD with outcomes by specific EGS operation (Supplementary Table 1). With non-CKD as reference*,* CKD 4–5 patients in all operative subtypes had a nearly 4–6-fold increased risk of developing AKI. ESRD increased costs by the largest margin on index hospitalization costs for those undergoing small bowel resection (*β* +$83,600, 95% CI $69,400–$97,800) and the lowest risk-adjusted cost burden on patients undergoing cholecystectomy (+$30,400, 95% CI $28,600–$32,200).

## DISCUSSION

Although CKD has been associated with adverse outcomes following a wide range of operations and elective abdominal surgery, limited data exist regarding its impact on outcomes following EGS. First, we found CKD to be associated with increased rates of risk-adjusted mortality and AE in a stepwise manner following EGS. Second, our study showed CKD to be associated with increased odds of several complications, including cerebrovascular, infectious, and AKI. Third, all grades of CKD were associated with increased hospitalization costs. ESRD was also associated with increased likelihood of nonhome discharge and odds of 90-day readmission. Several of these findings warrant further discussion.

In the present study, we found unadjusted rates of mortality to significantly rise as CKD severity increased. After stratifying by EGS operation, adjusted rates of mortality continued to remain high among CKD groups. Our results are consistent with prior work that associates ESRD with greater mortality following appendectomy and repair of perforated gastroduodenal ulcers [3,4] while extending this association to 4 additional common EGS operations. The observed trend in risk-adjusted mortality with increasing severity of CKD may be related to impaired homeostatic control mechanisms, platelet dysfunction, electrolyte imbalances, and oxidative stress secondary to diminished renal function. Similar to prior work, we found CKD to be associated with AE and specific complications [5,8,17,18], including a 1.2-fold increase in stroke and 3-fold increase in AKI. Thus, improvement in perioperative management of these patients and awareness of further deterioration of kidney function may improve postoperative recovery.

We also noted all grades of CKD to be associated with significantly higher hospitalization costs compared to others. These findings may reflect a greater intensity of care required to manage these patients, such as intensive care unit admission or in-hospital dialysis. In fact, other studies have found CKD to be attributable to $1,000–$65,000 in increased median costs among in-patient admissions [2,19]. In our study, patients with CKD had greater rates of nonhome discharge, further contributing to resource use after hospitalization. ESRD was associated with increased odds of 90-day readmissions similar to other studies [2,20,21]. These findings suggest that improved discharge planning and closer outpatient follow-up may minimize excess costs and reduce readmission rates.

The present work has several limitations due its retrospective design. As specific creatinine or glomerular filtration rates were unavailable in our database, we used validated codes for CKD stage to classify our groups within the study cohort. Similarly, we were unable to determine the duration of renal disease, which may affect clinical outcomes. Patient identifiers are not tracked across calendar years or in the outpatient setting, potentially contributing to underestimation of mortality and readmission rates. Comparisons of readmission rates across EGS subgroups are based on sampling and may not fully capture the true burden of CKD for higher-risk operations. Nonetheless, we used a contemporary national data set to study the impact of CKD on mortality, perioperative complications, and hospitalization costs and readmissions following 6 common EGS operations.

In conclusion, we found CKD to be associated with increased odds of mortality perioperative complications and higher index hospitalization costs. Moreover, ESRD was associated with increased odds of nonhome discharge and 90-day readmission. This nationally representative study builds on existing literature and provides insight to the greater risk of poor outcomes following EGS among those with CKD. These findings warrant additional research on interventions to improve surgical outcomes and reduce readmissions in patients with CKD that require EGS.

The following are the supplementary data related to this article.

Supplementary Table 1 Adjusted odds of complications by EGS operation. * indicates statistical significance at *P* < .05. ¥ indicates omission due to collinearity. Supplementary Table 1

## Author Contribution

Conceptualization: Vishal Dobaria, Zachary Tran, Joseph Hadaya, Yas Sanaiha, Peyman Benharash. Formal analysis: Vishal Dobaria, Joseph Hadaya, Arjun Verma. Investigation: Vishal Dobaria, Joseph Hadaya, Cory Lee, Arjun Verma, Yas Sanaiha, Peyman Benharash. Methodology: Vishal Dobaria, Joseph Hadaya, Cory Lee, Zachary Tran, Yas Sanaiha, Peyman Benharash. Project administration: Vishal Dobaria, Joseph Hadaya, Peyman Benharash. Resources: Peyman Benharash. Supervision: Joseph Hadaya, Peyman Benharash. Validation: Joseph Hadaya, Yas Sanaiha, Visualization: Peyman Benharash. Writing – original draft: Vishal Dobaria, Joseph Hadaya, Shannon Richardson, Arjun Verma, Cory Lee, Zachary Tran. Writing – review/editing: Vishal Dobaria, Joseph Hadaya, Shannon Richardson, Cory Lee, Yas Sanaiha, Peyman Benharash.

## Conflict of interest

None.

## Funding Source

None.

## References

[1] Chronic Kidney Disease Basics | Chronic Kidney Disease Initiative. https://www.cdc.gov/kidneydisease/basics.html.

[2] Sanaiha Y., Kavianpour B., Downey P. (2020). National study of index and readmission mortality and costs for thoracic endovascular aortic repair in patients with renal disease. Ann Thorac Surg.

[3] Smith M.C., Boylan M.R., Tam S.F. (2015). End-stage renal disease increases the risk of mortality after appendectomy. Surgery.

[4] Gross D.J., Chung P.J., Smith M.C. (2018). End stage renal disease is associated with increased mortality in perforated gastroduodenal ulcers. Am Surg.

[5] Liang C.C., Wang S.M., Kuo H.L. (2014). Upper gastrointestinal bleeding in patients with CKD. CJASN.

[6] Brakoniecki K., Tam S., Chung P. (2017). Mortality in patients with end-stage renal disease and the risk of returning to the operating room after common general surgery procedures. Am J Surg.

[7] Prowle J.R., EPY Kam, Ahmad T. (2016). Preoperative renal dysfunction and mortality after non-cardiac surgery. BJS.

[8] Jeong Y.S., Kim J., Kim D. (2021). Prediction of postoperative complications for patients of end stage renal disease. Sensors.

[9] Ozrazgat-Baslanti T., Thottakkara P., Huber M. (2016). Acute and chronic kidney disease and cardiovascular mortality after major surgery. Ann Surg.

[10] Hobson C., Ruchi R., Bihorac A. (2017). Perioperative acute kidney injury: risk factors and predictive strategies. Crit Care Clin.

[11] NRD overview https://www.hcup-us.ahrq.gov/nrdoverview.jsp.

[12] Hadaya J., Sanaiha Y., Juillard C. (2021). Impact of frailty on clinical outcomes and resource use following emergency general surgery in the United States. PLOS ONE.

[13] Elixhauser Comorbidity Software Version 3.7. https://www.hcup-us.ahrq.gov/toolssoftware/comorbidity/comorbidity.jsp.

[14] Using appropriate price indices for expenditure comparisons https://meps.ahrq.gov/about_meps/Price_Index.shtml.

[15] Tibshirani R. (1996). Regression shrinkage and selection via the Lasso. J R Stat.

[16] Klein D. (2021). MIMRGNS: Stata module to run margins after Mi estimate. https://econpapers.repec.org/software/bocbocode/S457795.htm.

[17] Havens J.M., Peetz A.B., Do W.S. (2015). The excess morbidity and mortality of emergency general surgery. J Trauma Acute Care Surg.

[18] Cloyd J.M., Ma Y., Morton J.M. (2014). Does chronic kidney disease affect outcomes after major abdominal surgery? Results from the National Surgical Quality Improvement Program. J Gastrointest Surg.

[19] Wang V., Vilme H., Maciejewski M.L. (2016). The economic burden of chronic kidney disease and end-stage renal disease. Semin Nephr.

[20] Muthuvel G., Tevis S.E., Liepert A.E. (2014). A composite index for predicting readmission following emergency general surgery. J Trauma Acute Care Surg.

[21] Kelley K.M., Collins J., Britt L.D. (2020). Readmission after emergency general surgery. Am J Surg.

