# Supplementary material for: Clinical and financial impact of chronic kidney disease in emergency general surgery operations
Source: Surg Open Sci. 2022 Jun 7;10:19–24. doi: 10.1016/j.sopen.2022.05.013 (PMC9283654; doi:10.1016/j.sopen.2022.05.013)
Supplement: Supplementary Table 1 — Adjusted odds of complications by EGS operation. * indicates statistical significance at P < .05. ¥ indicates omission due to collinearity. [file mmc2.docx]

**Supplemental Table 1** Adjusted Odds of Complications by EGS Operation. * indicates statistical significance at p<0.05. ¥ indicates omission due to collinearity

|  | ***Non-CKD*** | ***CKD 1-3*** | ***CKD 4-5*** | ***ESRD*** |
| --- | --- | --- | --- | --- |
|  |  |  |  |  |
| **Cholecystectomy** |  |  |  |  |
| Acute Kidney Injury | Ref | 3.00(2.86-3.14)* | 5.10(4.59-5.69)* | ¥ |
| Cardiac | Ref | 0.72(0.65-0.79)* | 0.85(0.70-1.05) | 0.99(0.85-1.16) |
| Cerebrovascular | Ref | 2.88(1.29-6.42)* | 1.34(0.18-10.7) | ¥ |
| Infectious | Ref | 0.97(0.93-1.02) | 0.98(0.87-1.10) | 0.94(0.86-1.02) |
| Respiratory | Ref | 0.77(0.71-0.82)* | 0.83(0.72-0.97)* | 1.01(0.90-1.13) |
| VTE | Ref | 0.74(0.57-0.95)* | 0.58(0.33-1.04) | 1.35(0.97-1.89) |
| **Appendectomy** |  |  |  |  |
| Acute Kidney Injury | Ref | 2.64(2.34-2.99)* | 4.59(3.52-5.96)* | ¥ |
| Cardiac | Ref | 0.89(0.72-1.11) | 1.74(1.21-2.50)* | 1.26(0.96-1.63) |
| Cerebrovascular | Ref | 1.29(0.34-4.87) | ¥ | 1.24(0.26-5.90) |
| Infectious | Ref | 0.90(0.79-1.03) | 0.96(0.73-1.26) | 1.32(1.12-1.55) |
| Respiratory | Ref | 0.72(0.62-0.84)* | 0.79(0.59-1.08) | 1.05(0.87-1.26) |
| VTE | Ref | 0.68(0.46-1.00) | 0.84(0.38-1.83) | 0.82(0.51-1.31) |
| **Perforation Repair** |  |  |  |  |
| Acute Kidney Injury | Ref | 2.47(2.05-2.98)* | 6.00(3.79-9.53)* | ¥ |
| Cardiac | Ref | 0.99(0.77-1.26) | 0.84(0.50-1.41) | 0.92(0.66-1.28) |
| Cerebrovascular | Ref | 3.72(0.52-26.6) | ¥ | ¥ |
| Infectious | Ref | 0.89(0.74-1.07) | 0.94(0.63-1.39) | 1.83(1.36-2.45) |
| Respiratory | Ref | 0.75(0.62-0.91)* | 0.65(0.43-0.97)* | 1.50(1.11-2.01)* |
| VTE | Ref | 1.04(0.69-1.56)* | 0.41(0.14-1.24) | 1.10(0.65-1.04) |
| **Lysis of Adhesions** |  |  |  |  |
| Acute Kidney Injury | Ref | 2.48(2.05-2.99)* | 5.38(3.59-8.04)* | ¥ |
| Cardiac | Ref | 0.86(0.61-1.22) | 1.67(0.93-2.99) | 0.91(0.58-1.42) |
| Cerebrovascular | Ref | 3.89(0.28-50.0) | ¥ | ¥ |
| Infectious | Ref | 0.84(0.69-1.00) | 1.77(1.22-2.59)* | 0.73(0.58-0.93)* |
| Respiratory | Ref | 0.77(0.61-0.97)* | 1.41(0.92-2.16) | 1.02(0.77-1.35)* |
| VTE | Ref | 0.24(0.08-0.68)* | 0.47(0.55-4.09) | 2.29(1.04-5.04)* |
| **Small Bowel Resection** |  |  |  |  |
| Acute Kidney Injury | Ref | 2.42(2.22-2.63)* | 4.02(3.32-4.87)* | ¥ |
| Cardiac | Ref | 0.78(0.68-0.89)* | 0.97(0.76-1.24) | 1.23(1.05-1.45)* |
| Cerebrovascular | Ref | 0.22(0.03-1.67) | 1.06(0.14-8.24) | 0.56(0.07-4.32) |
| Infectious | Ref | 0.91(0.84-0.99)* | 0.82(0.69-0.98)* | 1.77(1.56-2.00)* |
| Respiratory | Ref | 0.76(0.69-0.83)* | 0.92(0.76-1.11) | 1.66(1.47-1.88)* |
| VTE | Ref | 0.76(0.59-0.97)* | 0.58(0.34-0.99)* | 1.11(0.82-1.50) |
| **Large Bowel Resection** |  |  |  |  |
| Acute Kidney Injury | Ref | 2.53(2.37-2.70)* | 4.60(3.97-5.32)* | ¥ |
| Cardiac | Ref | 0.88(0.80-0.97) | 0.99(0.83-1.19) | 1.26(1.12-1.41) |
| Cerebrovascular | Ref | 0.62(0.24-1.62) | 0.60(0.08-4.44) | 0.89(0.27-2.96) |
| Infectious | Ref | 0.90(0.84-0.96)* | 1.08(0.94-1.23) | 2.03(1.84-2.24)* |
| Respiratory | Ref | 0.84(0.78-0.90)* | 1.02(0.88-1.17) | 2.02(1.83-2.22)* |
| VTE | Ref | 0.67(0.57-0.81)* | 0.72(0.52-1.01) | 1.11(0.90-1.36) |
